# Exploiting tertiary lymphoid structures gene signature to evaluate tumor microenvironment infiltration and immunotherapy response in colorectal cancer

**DOI:** 10.3389/fonc.2024.1383096

**Published:** 2024-05-23

**Authors:** Zhu Xu, Qin Wang, Yiyao Zhang, Xiaolan Li, Mei Wang, Yuhong Zhang, Yaxin Pei, Kezhen Li, Man Yang, Liping Luo, Chuan Wu, Weidong Wang

**Affiliations:** ^1^ Department of Oncology, School of Clinical Medicine, Southwest Medical University, Luzhou, China; ^2^ Department of Radiation Oncology, Sichuan Clinical Research Center for Cancer, Sichuan Cancer Hospital & Institute, Sichuan Cancer Center, Affiliated Cancer Hospital of University of Electronic Science and Technology of China, Chengdu, China; ^3^ Department of Pathology, QuXian People’s Hospital, Dazhou, China; ^4^ School of Medicine, University of Electronic Science and Technology of China, Chengdu, China

**Keywords:** tertiary lymphoid structures, colorectal cancer, tumor microenvironment, prognostic, immunotherapy

## Abstract

**Background:**

Tertiary lymphoid structures (TLS) is a particular component of tumor microenvironment (TME). However, its biological mechanisms in colorectal cancer (CRC) have not yet been understood. We desired to reveal the TLS gene signature in CRC and evaluate its role in prognosis and immunotherapy response.

**Methods:**

The data was sourced from The Cancer Genome Atlas (TCGA) and the Gene Expression Omnibus (GEO) databases. Based on TLS-related genes (TRGs), the TLS related subclusters were identified through unsupervised clustering. The TME between subclusters were evaluated by CIBERSORT and xCell. Subsequently, developing a risk model and conducting external validation. Integrating risk score and clinical characteristics to create a comprehensive nomogram. Further analyses were conducted to screen TLS-related hub genes and explore the relationship between hub genes, TME, and biological processes, using random forest analysis, enrichment and variation analysis, and competing endogenous RNA (ceRNA) network analysis. Multiple immunofluorescence (mIF) and immunohistochemistry (IHC) were employed to characterize the existence of TLS and the expression of hub gene.

**Results:**

Two subclusters that enriched or depleted in TLS were identified. The two subclusters had distinct prognoses, clinical characteristics, and tumor immune infiltration. We established a TLS-related prognostic risk model including 14 genes and validated its predictive power in two external datasets. The model’s AUC values for 1-, 3-, and 5-year overall survival (OS) were 0.704, 0.737, and 0.746. The low-risk group had a superior survival rate, more abundant infiltration of immune cells, lower tumor immune dysfunction and exclusion (TIDE) score, and exhibited better immunotherapy efficacy. In addition, we selected the top important features within the model: *VSIG4*, *SELL* and *PRRX1*. Enrichment analysis showed that the hub genes significantly affected signaling pathways related to TLS and tumor progression. The ceRNA network: *PRRX1*-miRNA (*hsa-miR-20a-5p*, *hsa-miR-485–5p*) -lncRNA has been discovered. Finally, IHC and mIF results confirmed that the expression level of *PRRX1* was markedly elevated in the TLS- CRC group.

**Conclusion:**

We conducted a study to thoroughly describe TLS gene signature in CRC. The TLS-related risk model was applicable for prognostic prediction and assessment of immunotherapy efficacy. The TLS-hub gene *PRRX1*, which had the potential to function as an immunomodulatory factor of TLS, could be a therapeutic target for CRC.

## 1 Introduction

CRC is among one of the most prevalent cancers worldwide. Recent surveys have shown that CRC accounted for 10% of new cancer cases and deaths globally (1, 2). Notwithstanding progress in surgical techniques, chemotherapy, and radiation therapy, a substantial number of patients experience recurrence or metastasis. Immunotherapy has opened new avenue for treatment, particularly for patients with specific genetic mutations like deficient mismatch repair (dMMR) and microsatellite instability-high (MSI-H). Clinical trials, including KEYNOTE-164, CheckMate 142 and others, have shown that the use of programmed cell death protein-1 (PD-1) blockade therapy leads to long-lasting response, especially in advanced-stage patients with MSI-H (3–5). However, immunotherapy is not universally effective; it benefits only a subset of patients and can cause unique side effects, such as autoimmune reactions. The effectiveness of immunotherapy largely depends on the TME and genetic profiles. According to reports, TLS was positively correlated with the expression of immune-related genes in CRC, such as programmed cell death ligand-1 (PD-L1) (6). This suggests that TLS may enhance the effectiveness of immune checkpoint blockade (ICB) therapies by promoting the infiltration of immune cells and remodeling TME.

TLS, also referred to ectopic lymphoid organizations (ELO), serves as a site for immune cells recruitment, activation, and proliferation (7–9). TLS resemble lymph nodes to some extent but with unique features. For example, TLS lack capsules and afferent lymphatic vessels, which distinguishes them from traditional lymph nodes. TLS contains structured zones of immune cells, such as the T cell zone and the B cell germinal center (GC) (10). In summary, TLS is comprised of immune cells, stromal cells, and specialized vascular components (11, 12). TLS has a vital function in local immune responses. Research on TLS is currently flourishing in the fields of cancer, chronic inflammation, and autoimmune diseases (13).

Accumulating evidences suggest that TLS generally associated with favorable prognosis and better immunotherapy efficacy in melanoma, breast cancer and so on (11, 14–16). Patients with TLS tend to have improved OS rates compared to those lacking TLS. TLS can convert an immune cold tumor to a hot tumor by increasing the recognition and clearance of the host immune system to tumors (17). In addition, the latest study found that combining immunotherapies with strategies to promote TLS formation or reorganization may enhance the effectiveness of immunotherapy. Strategies may include using specific cytokines or chemokines to recruit immune cells to TLS.

Despite progress, there remains an incomplete understanding of the precise molecular mechanisms governing TLS growth and maturity. There are still doubts about the interactions between TLS and the tumor immune system. Therefore, our study integrated TRGs and downloaded data from TCGA database to classify patients into two subclusters and develop a novel prognostic model. We also investigated the connection between TLS-hub genes and its molecular mechanisms.

## 2 Materials and methods

### 2.1 Data collection and preparation

The data, including gene sequencing data and corresponding clinical data, were obtained from the TCGA and GEO databases. TCGA-CRC dataset functioned as a discovery set for marker selection and model construction, consisting of 558 samples. Samples with survival times under 30 days were removed. GEO datasets GSE38832 and GSE17537 served as external validation sets to assess the robustness of the model. IMvigor210 immunotherapy cohort was obtained from the original article (18). Clinical information for all samples were detailed in **Supplementary Table 1**.

### 2.2 Establish TLS-related molecular subclusters and evaluate the TME

The mutation landscapes of CRC patients were analyzed by the “maftool” R package (19). Unsupervised clustering, via the “ConsensusClusterPlus” R package, segmented patients into two subclusters (20). This segmentation was guided by maximizing intergroup variances and minimizing intragroup variances. The immune infiltration environment in each patient was assessed using CIBERSORT and xCell (21, 22). XCell was also utilized to compare the environment scores in different groups.

### 2.3 Develop the TLS-related risk model, estimate its prognostic value, and develop a comprehensive nomogram

Using the “DESeq2” package, we obtained differentially expressed genes (DEGs) comparing two clusters, tumor and normal. The Lasso-Cox regression was applied to filter out DEGs that are associated with OS, reduce dimensions, and construct the prognostic model. Patients were classified into two risk groups by the risk score, which was calculated as: $Risk\;Score=\sum_{i=1}^{14}Coe_{f}^{i}*Expr_{i}$ *Coef_i_* denotes the estimated regression coefficient, while *Expr_i_* denotes the gene’s expression level. The model’s predictive efficiency and accuracy were evaluated via the “survival”, “survminer”, “timeROC”, and “pheatmap” R packages. MSI status was obtained from the “cBioPoralData” R package. According to the results of multivariate Cox regression, we plotted the forest plot and nomogram.

### 2.4 Identify TLS-related hub genes, analyze its molecular mechanism and enrichment pathways

The 14 model genes were ranked based on the feature importance calculated through “randomForest” package. All feature importance was normalized to range from 0 to 1. Genes with importance exceeding 0.75 were classified as TLS-related hub genes. To comprehensively analyze the biological pathways of TLS-related genes, a dual approach involving gene set enrichment analysis (GSEA) and gene set variation analysis (GSVA) were used. Gene Ontology (GO), Kyoto Encyclopedia of Genes and Genomes (KEGG), and hallmark annotation were conducted with the “clusterProfiler” and “GSVA” packages (23, 24). The visualization of results using the “ggplot2”.

### 2.5 Estimation of TLS existence in human CRC tissue samples

Human CRC tissue samples were collected from QuXian People’s Hospital, including 20 TLS+ tissue samples and 10 TLS- tissue samples (initial diagnosis of CRC, denial of other tumors, unaccepting of neoadjuvant therapy and complete clinical data). Clinical information for all samples was detailed in **Supplementary Table 3**. The research received approval from the Ethics Committee of the QuXian People’s Hospital. Two pathologists independently evaluated the TLS existence in all CRC samples. Questionable results were then co-reviewed for an agreed annotation. TLS were classified into three stages based on maturity: (1) early TLS (compact clumps of lymphocytes without segregated T and B cell zones), (2) primary follicle-like TLS [(have follicular dendritic cell (FDC), lack of GC)] and (3) secondary follicle-like TLS (have an active GC) (25). Samples with TLS were categorized as TLS+ group, while others were deemed TLS- group.

### 2.6 Using mIF to characterize TLS and related hub gene

Firstly, slides were deparaffinized and placed in the citrate-phosphate buffer (heat them in a microwave at low heat for 15 min). Circled the tissue with an immunohistochemical pen to block endogenous peroxidase. Secondly, incubated the slides in hydrogen peroxide water at room temperature and avoid light for 25 min. Added BSA buffer dropwise onto the slides and sealed for 30 min. After removing the BSA buffer, we added the prepared primary antibody (CD3: ab16669, Abcam, Britain, 1:1000) dropwise and incubated the slides overnight at 4 °C in a wet box. Next, the slides were washed, and horseradish peroxidase (HRP) labelled secondary antibody (Goat anti-mouse/rabbit IgG H&L, RCB054, RecordBio, China) were dripped onto the slides. Tyramide signal amplification (TSA) buffer was used to visualize each biomarker. Dripped corresponding TSA dye onto the slides and incubated them at room temperature in the dark for 10 min to stain the antibody. Repeated the previous steps using different primary antibodies (CD20: ab78237, Abcam, 1:1000; CD21: ab315160, Abcam, 1:1000) and switched different TSA dyes. Finally, stained nucleus with DAPI and coverslipped anti-fluorescence quenching to preserve fluorescence. The slides were digitized using OLYMPUS (OlyVIA, Japan).

Next, we performed IHC to examine the *PRRX1* expression level in TLS+/- CRC formalin-fixed paraffin-embedded (FFPE) samples, using *PRRX1* antibody (ab211292, Abcam, 1:100). Tissues were scored according to the product of stained area percentage (0 = 0%, 1 = 1%–25%, 2 = 26%–50%, 3= 51%–75%, and 4 = more than 75%) and the staining intensity scale [ranged from 0 (no staining) to 3 (strong staining), measured by Halo]. The IHC score for all samples was detailed in **Supplementary Table 3**.

### 2.7 Statistical analysis

The data analysis was performed using R software version 4.2.2. For categorical data, the Chi-square test and Fisher’s exact test were used to assess differences between groups. For continuous data, if the data are normally distributed and have equal variances, an independent samples T-test was used; if the data do not follow a normal distribution, the Mann-Whitney U test was used. Spearman’s correlation analysis was used to identify the relationship between two variables. The threshold for statistical significance was set at P< 0.05 (two-tailed).

## 3 Results

### 3.1 The genetic and variation characteristics of TRGs in CRC

**Figure 1:**
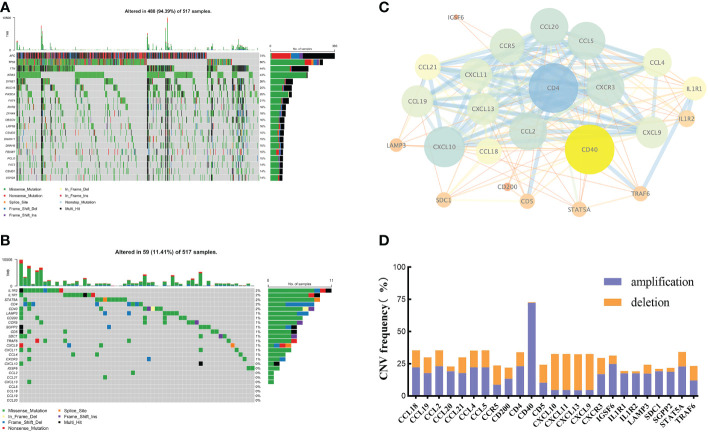
The genetic and variation landscapes of TCGA-CRC dataset. **(A)** Somatic mutation of the top 20 genes in CRC. **(B)** Somatic mutation of 25 TRGs. **(C)** PPI network of TRGs. The size and color of the nodes represented the degree of interaction. The edge size and the color showed the combined score of interaction. **(D)** CNV frequency of TRGs.

By integrating current studies and reviews, we identified 42 TRGs, of which 25 were expressed in TCGA-CRC dataset (**Supplementary Table 2**). Among 517 CRC patients, 94.39% (488) experienced somatic mutations. *APC* and *TP53* had the highest mutation frequencies (**Figure 1A**). We further analyzed the somatic mutation pattern of TRGs (**Figure 1B**). 11.41% (59) of patients experienced somatic mutations, predominantly missense mutations.*IL1R1* and *IL1R2* exhibited the highest mutation frequencies, while *CD4* and *LAMP3* primarily underwent frame shift deletions. Using STRING (V12.0), a protein-protein interaction (PPI) network was constructed (**Figure 1C**). GISTIC2.0 was used to calculate the copy number variations (CNV). The results showed that copy number amplification (including homozygous and heterozygous mutations) was the major mutation (**Figure 1D**).

### 3.2 Clinicopathological characteristics and immune cell infiltration of TLS related subclusters

**Figure 2:**
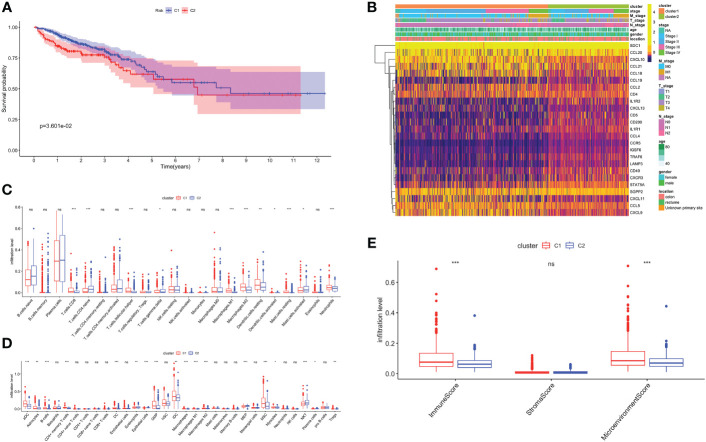
Biological characteristics of two TLS related subclusters. **(A)** Kaplan-Meier survival analysis of C1 and C2. **(B)** TRGs expression and clinicopathological characteristics heatmap. Immune cell infiltration patterns by Cibersort **(C)** and xCell **(D)**. **(E)** The immune, stromal and microenvironment scores between two clusters. ns, not significance, *p< 0.05, **p< 0.01, ***p< 0.001.

Unsupervised consensus clustering based on TRGs categorized CRC patients into two molecular subclusters. The consensus clustering results indicated that the optimal clustering number was 2 (**Supplementary Figures 1A–C**). Principal component analysis (PCA) proved the optimal division into two clusters, C1 and C2 (**Supplementary Figure 1D**). The Kaplan-Meier analysis demonstrated a statistically difference in C1 and C2 (P<0.01, **Figure 2A**). The prognosis for C1 was more favorable compared to C2. Meanwhile, we examined the clinicopathological characteristics of two subclusters. C1 had a higher proportion of males, N0, M0, and stage I/II (**Supplementary Figures 1D–I**).

Next, we analyzed the TRGs expression profiles of two subclusters in the TCGA-CRC dataset (**Figure 2B**). We found C1 had higher expression levels of *CCL18*, *CCL21*, *CXCL10* and *CXCL11*. C2 was characterized by the remarkable enrichment in *SDC1* and *CCL20*. In general, TRGs were more highly expressed in C1 than C2. Immune infiltrating cells are a crucial component of TME. We explored the differences in TME infiltration between two subclusters. (**Figures 2C, D**). B cells, CD8+ T cells, follicular helper T (T_FH_) cells, activated dendritic cells (DC), neutrophils and granulocyte-macrophage progenitor (GMP) cells were higher in C1, while naive CD4+ T cells and natural killer T (NKT) cells were higher in C2. Both macrophage M1 and macrophage M2 had higher infiltration levels in C1. Besides, we further noticed that C1 correlated positively with higher immune and microenvironment scores, while there was no difference between two subclusters in stroma scores (**Figure 2E**).

### 3.3 Biological functional enrichment analysis of TLS molecular subclusters

**Figure 3:**
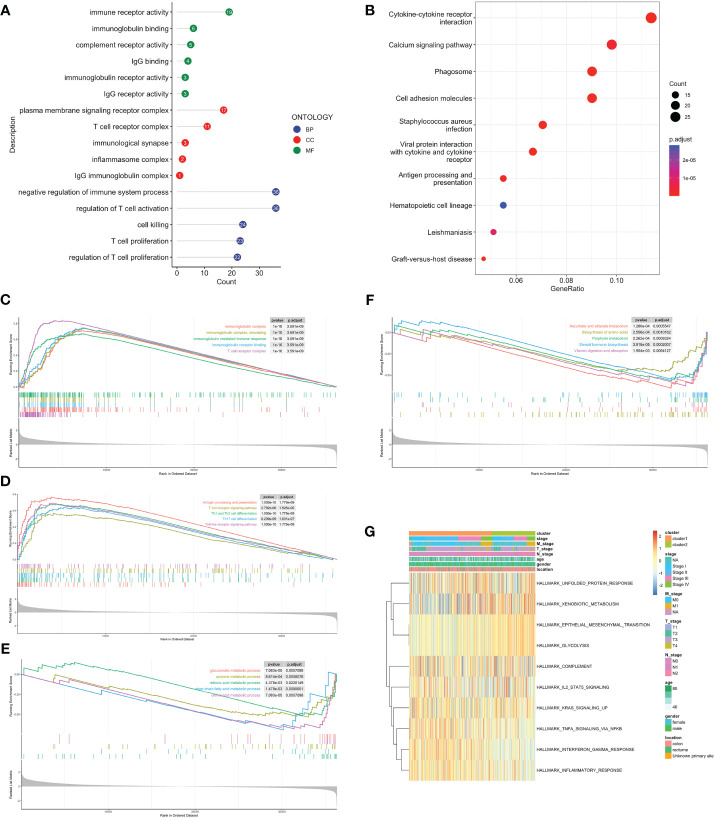
Biological functional enrichment analysis. GO **(A)** and KEGG **(B)** enrichment analyses based on DEGs. GSEA of C1 by GO **(C)**, KEGG **(D)**. GSEA of C2 by GO **(E)**, KEGG **(F)**. **(G)** GSVA of two clusters by Hallmark database.

Volcano map was used to demonstrate DEGs between C1 and C2, with 639 genes upregulated in C1 and 86 in C2 (**Supplementary Figure 2A**). Firstly, we performed over-representation analysis (ORA) to determine the biological behavior behind these DEGs. The GO enrichment analysis revealed that immune receptor activity enrichment in molecular function (MF); plasma membrane signaling receptor complex and immunological synapse enrichment in cellular component (CC); T cell proliferation and activation enrichment in biological process (BP) (**Figure 3A**). The KEGG analysis indicated predominantly enrichment in cytokine-cytokine receptor interaction, calcium signaling pathway, and cell adhesion molecules (**Figure 3B**). GSEA showed that C1 was enriched in immune response, activation, and antigen presentation, including immunoglobulin mediated immune response, Th1 and Th2 cells differentiation, and Toll-like receptor signaling pathway (**Figures 3C, D**). Metabolic processes and biosynthesis related pathways were enriched in C2, such as glucuronate metabolic process, and biosynthesis of amino acids and steroid hormone (**Figures 3E, F**). GSVA showed differentially active signaling pathways and immune responses between two clusters (**Figure 3G**). Taken together, this suggested that TLS-enriched subcluster, C1, may inhibit tumor progression by activating non-specific anti-tumor immune responses. While TLS-depleted subcluster, C2, was for hypermetabolic and immunosuppression state.

### 3.4 Development and verification of the TLS-related risk model

To further analyze and quantify the TLS characteristics, a TLS-related prognostic model was established. TCGA-CRC dataset served as the training set, while GSE38832 and GSE17537 were utilized for the external validation. DEGs between tumor and normal samples were measured with the “DESeq2” (**Supplementary Figure 2B**). There were 508 intersection genes left (**Supplementary Figure 2C**, **Supplementary Table 2**). After univariable Cox regression analysis, we found 43 OS-related DEGs. Ultimately, 14 genes were selected for the risk model: *CAVIN2*, *CCL19/21*, *CD8A*, *CXCL5*, *FHL1*, *IGHG1*, *MMP1*, *MRC1*, *NEXN*, *MOTUM*, *PRRX1*, *SELL* and *VSIG4* (**Supplementary Figures 2D, E**). The model genes displayed a distinct expression pattern between two groups (**Supplementary Figure 2F**).

**Figure 4:**
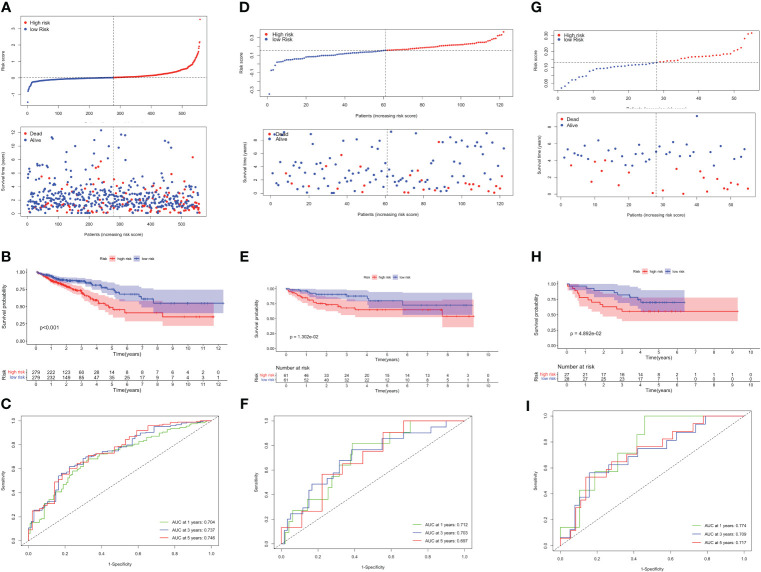
Validation of the TLS-related prognostic model in TCGA-CRC, GSE38832 and GSE17537 datasets. **(A)** Risk score distribution (up) and survival status (down) between high- and low-risk groups. **(B)** Kaplan–Meier survival analysis. **(C)** Time-dependent ROC analysis of risk scores. Risk score distribution and survival status **(D)**, Kaplan–Meier survival analysis **(E)**, and Time-dependent ROC analysis **(F)** in GSE38832. Risk score distribution and survival status **(G)**, Kaplan–Meier survival analysis **(H)**, and Time-dependent ROC analysis **(I)** in GSE17537.

Based on the optimum cutoff value, patients were divided into two risk groups. Kaplan-Meier curves indicated lower mortality and better prognosis in the low-risk group (P<0.001, **Figures 4A, B**). Furthermore, the AUC revealed the 1-, 3-, and 5-year survival rates were all above 0.7, which proved the accuracy of this model was wonderful (**Figure 4C**). External validation datasets GSE38832 and GSE17537 also demonstrated the model’s excellent prognostic performance (**Figures 4D–I**).

### 3.5 Immune infiltration of TLS-related risk model

**Figure 5:**
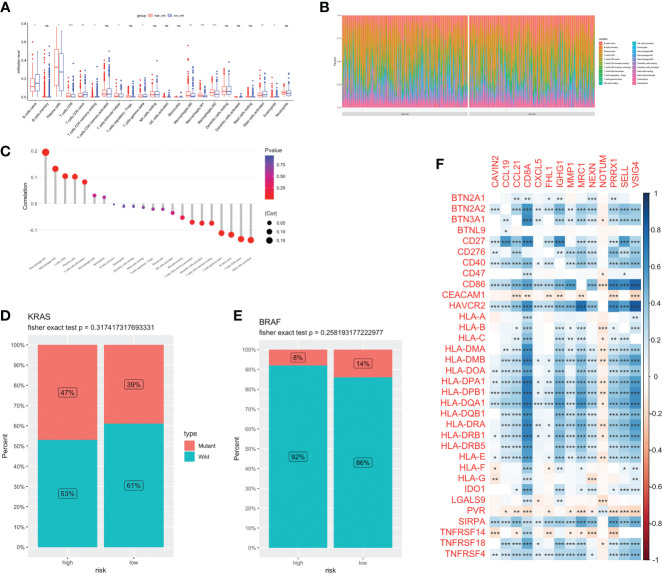
Immune infiltration and TME of low-and high-risk groups. Immune cell infiltration **(A)** and percentage **(B)** between two groups. **(C)** Correlation analysis between risk score and infiltration level. Risk score and CRC molecular features: KRAS **(D)** and BRAF **(E)**. **(F)** The correlations between immune genes and model genes. ns, not significance, *p< 0.05, **p< 0.01, ***p< 0.001.

Different kinds of immune cell infiltration were estimated using CIBERSORT (**Figure 5A**). Plasma cells, macrophages and follicular helper T cells were higher in high-risk group. Additionally, we estimated the proportion of each cell in TME (**Figure 5B**). There were remarkably positive associations between risk score and CD8+ T cells, CD4+ naive T cells, macrophages and activated mast cells (P<0.01, **Figure 5C**). Further analysis revealed that there were more patients with KRAS mutations and fewer patients with BRAF mutations in the high-risk group (no statistically significant, **Figures 5D, E**). Next, we estimated the association between immune checkpoint genes and 14 model genes (**Figure 5F**). We found that the vast majority of model genes were positively correlated with immune related genes, except NOTUM.

### 3.6 Predict the efficacy of immunotherapy

Our above findings demonstrated a potential correlation between risk score and immunotherapy response. The low-risk group had higher stroma, immune, and TIME scores (**Supplementary Figures 3A–C**). TIDE score has been extensively employed to assess the likelihood of immune evasion and resistance to immunotherapy. We discovered the high-risk group had a much higher TIDE score (**Supplementary Figure 3D**). A higher TIDE score was linked to worse ICB effectiveness as well as shorter OS with ICB therapies. The IMvigor210 dataset, involving patients treated with atezolizumab, was utilized to validate these observations. In IMvigor210, low-risk patients had a greater prognosis and higher disease control rate (p<0.05, **Supplementary Figures 3E, F**).

### 3.7 Construction of a comprehensive nomogram to predict prognosis

**Figure 6:**
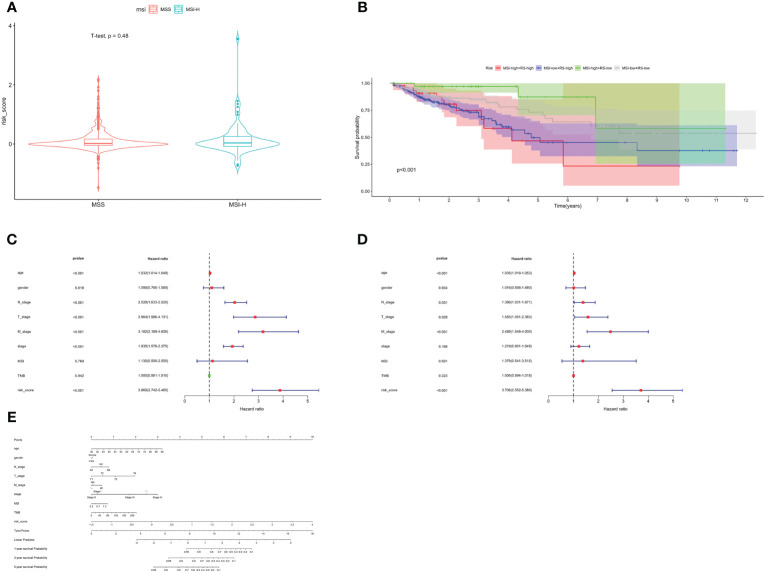
Stratified survival analysis and nomogram. **(A)** The relationship between risk score with different MSI status. **(B)** Stratified survival analysis combined with MSI status and risk score. Univariate Cox **(C)** and multivariate Cox **(D)** regression analyses. **(E)** The nomogram containing age, gender, T, N, M, stage, TMB and risk score.

MSI status was used to be an effective predictor for patients receiving ICB treatment. Typically, patients with high level of MSI were classified as MSI-High, indicating genomic instability resulting from repeated microsatellite expansions or contractions. Conversely, patients with low or no MSI were classified as microsatellite instability-low (MSI-L)/Microsatellite Stability (MSS). We analyzed the relationship between patients with different MSI status and risk score, and found no significant correlation (**Figure 6A**). Then, we combined the risk score with MSI status for stratified survival analysis (**Figure 6B**). Investigations indicated that MSI-H and low-risk scores patients had the best prognosis, followed by those with MSI-L and low-risk scores. Univariate and multivariate Cox regression confirmed the risk score as an independent prognostic factor for CRC (HR=3.706, 95%CI=2.552–5.380, **Figures 6C, D**). Exploiting these Clinicopathological characteristics, a new-type nomogram was constructed to assess the clinical prognostic more accurately (**Figure 6E**).

### 3.8 TLS-related hub gene identification

Random forest analysis was used to determine the feature importance of 14 model genes, among which three genes—*VSIG4*, *SELL* and *PRRX1*—stood out with importance scores exceeding 0.75 (**Supplementary Figure 4A**). In the high-risk group, the expression level of *VSIG4*, *SELL* and *PRRX1* were higher (**Supplementary Figure 4B**). Correlation analysis revealed that the vast majority of model genes were significantly positively correlated with hub genes (**Supplementary Figure 4C**). We conducted a further study to explore the correlation between the expression of TLS-related hub genes and the infiltration of immune cells in TME (**Supplementary Figure 4D**). *VSIG4*, *SELL* and *PRRX1* exhibited a positive relationship with macrophages M2 and neutrophils.

### 3.9 Explore the potential molecular mechanisms of TLS-related hub genes

**Figure 7:**
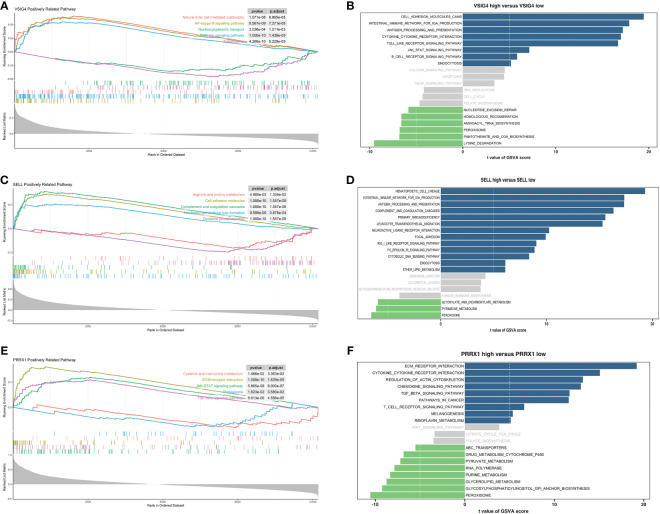
Enrichment analyses of *VSIG4*, *SELL* and *PRRX1*. **(A)** GSEA of *VSIG4*. **(B)** GSVA of *VSIG4*. **(C)** GSEA of *SELL*. **(D)** GSVA of *SELL*. **(E)** GSEA of *PRRX1*. **(F)** GSVA of *PRRX1*.

GSEA and GSVA were conducted to investigate the molecular mechanisms and immune functions of the three hub genes. Results indicated that *VSIG4* was positively correlated with signaling pathways such as NF-kappa B signaling pathways, PI3K-AKT signaling pathways, and cytokine-cytokine receptor interaction (**Figures 7A, B**). High expression of *SELL* primarily enriched in the intestinal immune network for IGA production, oxidative phosphorylation, arginine and proline metabolism, Fc epsilon signaling pathway, and others (**Figures 7C, D**). GSEA of *PRRX1* subsequently uncovered that *PRRX1* was significantly linked to epithelial-mesenchymal transition (ECM) -receptor interaction, proteasome and TGF-beta signaling pathway (**Figure 7E**). High expression of *PRRX1* was mainly enriched in riboflavin metabolism, regulation of actin cytoskeleton, and others (**Figure 7F**). The low expression of *PRRX1* was enriched in pyruvate metabolism, purine metabolism, peroxisome, etc. In addition, *PRRX1* may serve as a prominent regulatory factor in tumorigenesis and progression.

### 3.10 Identification of a hub gene related ceRNA network

**Figure 8:**
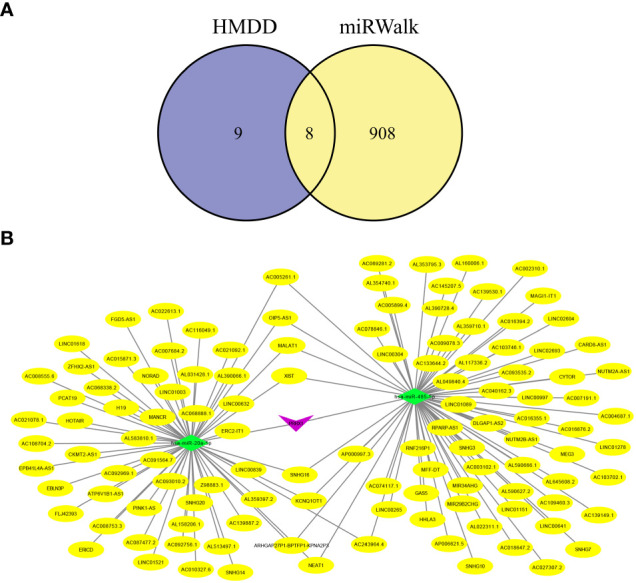
Hub genes-related ceRNA network analysis. **(A)** Intersection miRNAs of HMDB and miRWalk databases. **(B)** A hub genes-related ceRNA network of mRNA (*PRRX1*)-miRNA (hsa-miR-485–5p, hsa-miR-20a-5p)-lncRNA.

Increasing evidence has shown that the mutual regulatory patterns between mRNA, lncRNA, miRNA, and downstream target genes are intimately related to tumors. Our study exploited the Human MicroRNA Disease Database (HMDD) and miRNA Walkthrough (miRWalk) databases to identify the miRNA and lncRNA associated with CRC. Excluding interactions unrelated to disease, only two mRNA (*SELL*, *PRRX1*) and eight miRNA were retained (**Figure 8A**). Secondly, those mRNA-miRNA interactions were validated in the ENCORI database, *hsa-miR-485–5p* and *hsa-miR-20a-5p* regulated *PRRX1* were discovered. Utilizing Cytoscape, we constructed a ceRNA network, including 1 mRNA, 2 miRNA and 127 lncRNA (56 for *hsa-miR-20a-5p*, 71 for *hsa-miR-485–5p*) interaction pairs (**Figure 8B**).

### 3.11 Experimental validation

**Figure 9:**
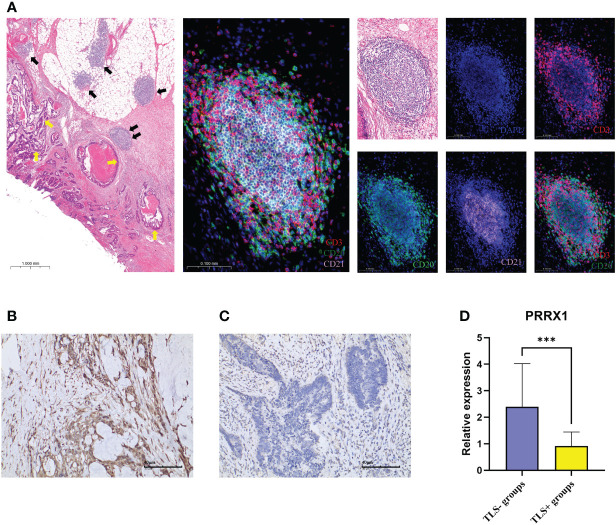
mIF and IHC characterized TLS and PRRX1 expression in CRC. **(A)** Representative images of secondary follicle-like TLS. The yellow arrows in the left image represented tumor tissue, the black arrows represented TLS. The TLS pointed by the two black arrows indicated the enlarged TLS in the subsequent image. The images contained H&E staining, mIF staining, and single IF under each marker (DAPI, CD3, CD20, CD21). IHC images (×200) of PRRX1 expression in the TLS- group **(B)** and TLS+ group **(C)**. **(D)** Differences in the expression of PRRX1 between TLS+/- groups. ***p< 0.001.

Using IHC to assess the *PRRX1* expression in the TLS+/- groups. Firstly, we observed TLS images through hematoxylin-eosin (H&E) and mIF (**Figure 9A**). The lymphocyte aggregates containing CD3+T cells, CD20+B cells, and CD21+FDCs that form corresponding regions are considered secondary follicle-like TLS, also known as mature TLS. To further validate the expression of *PRRX1*, we performed IHC on TLS+/- CRC tissues (**Figures 9B, C**). *PRRX1* was significantly higher in the TLS- group (**Figure 9D**). Through these above findings, we speculate that *PRRX1* may be a negative regulator of TLS and play a crucial role in tumor metastasis and immunocompetence.

## 4 Discussion

With the continuous development of anti-tumor therapy, immunotherapy has gradually become a common treatment method in clinical practice (26, 27). However, effective biomarkers to identify potential beneficiaries remain limited. PD-L1 and tumor mutation burden (TMB) are considered to be insufficient. Some patients receiving ICB treatments do not derive benefit, mainly due to the heterogeneity of TME and the complexity of immune mechanisms (28). TLS, as a particular component of TME, has been proven to independently predict the response to ICB, regardless of the PD-L1 expression and MSI status (15, 17, 29). In this research, we conducted a detailed analysis of the variations in immune infiltration and potential mechanisms caused by TLS, and developed a novel model for evaluating the prognosis and the effectiveness of immunotherapy in CRC.

Firstly, we integrated a large number of reviews and articles to recruit TRGs. TRGs including various TLS signatures relevant to CRC, melanoma, breast cancer, gastric cancer, ovarian cancer, soft tissue sarcoma, and others (13, 30–37). We initially investigated the expression and mutation patterns of TRGs in CRC. *IL1R1* and *IL1R2* had the highest mutation frequencies, *CD40* and *IGSF6* had the highest CNV amplification mutation frequencies. CD40 is a member of TNF receptor superfamily. The high mutation rate of CD40 is related to its structural changes and TRAF (TNF-α Receptor Associated Factor)-binding domain exposure induced by a residue mutation of its ligand CD40L. CD40-CD40L interaction promotes GC formation and maintenance, and Th1 (T helper 1) response conversion (38).

Unsupervised consensus clustering, as one of the methods of consensus clustering, can be used to distinguish various subclusters. According to TRGs, 2 TLS related subclusters (TLS-enriched/TLS-depleted) were identified. Survival analysis revealed that C1 had a more favorable prognosis. We further analyzed the immune infiltration patterns between two clusters. The abundance of B cells, CD8+ T cells, T_FH_ cells, neutrophils and DC were higher in C1. Studies by Qin F et al. indicated that anti-TGF-β facilitates neutrophils recruitment and polarizes neutrophils towards an anti-tumor phenotype (N1) in CRC (39). Meanwhile, tumor infiltrating neutrophils can exert the role of antigen-presenting cells and heighten T cell proliferation (40). T_FH_ cells are usually accompanied by high *CXCR5*, *CD40*, and *CXCL13* expression. *CXCL13*/*CXCR5* signaling axis is a critical signaling pathway for TLS formation (41). Research has shown that T_FH_ controls the proliferation of B cells in GC (42). Patients with a substantial fraction of T_FH_ cells usually have a more favorable clinical outcome (33). The GC with *CD20*+ B cells is considered a mature feature of TLS. B cells has the ability to generate antibodies and also enhance cytotoxic CD8+ T cell response by secreting various cytokines in TME, such as interleukin-2 (IL-2) and interferon-γ (IFN-γ) (43, 44). Various studies demonstrated a strong correlation between B cells, especially those in TLS, and the efficacy of immunotherapy (45, 46). Inducing the formation and maturation of TLS by enhancing B cell expression may improve the treatment response rates and OS in CRC. Immune and environment scores also indicate that C1 has an inflammatory immune phenotype and stands in an immune-activated state. Thereby, we infer that C1 is the TLS-enriched cluster, while C2 is the TLS-depleted cluster.

In addition, a prognostic risk model related to TLS was developed. The AUC values for OS at 1–3-5 years were 0.704, 0.737, and 0.746, as well. The external validation datasets GSE38832 and GSE17537 also demonstrated their excellent prognostic ability. Next, we further analyzed the immune infiltration landscapes, molecular mutation features and immunotherapy efficacy of the two risk groups. Based on risk score, TMB and other clinical features, we constructed a new-type nomogram to comprehensively evaluate the prognosis.

According to the random forest analysis, we selected TLS-related hub genes in the model: *VSIG4*, *SELL* and *PRRX1*. *VSIG4*, fully defined as V-set and immunoglobulin domain-containing 4, is a complement receptor of the B7 immunoglobulin superfamily. *VSIG4* is mainly expressed in macrophages. *VSIG4*-expressing tumor-associated macrophages (TAMs) can inhibit tumor-specific CD8+ T cell proliferation and cytokines production, which function as a suppressor of anti-tumor immune response (47, 48). Recent studies indicates that *VSIG4* could activate the PI3K-AKT-STAT3 pathway, upregulating PDK2 and thereby inhibiting mitochondrial pyruvate metabolism and mitochondria ROS secretion (49). The relationship between *VSIG4* and poor prognosis has been confirmed in lung cancer (50), ovarian cancer (51), and glioma (52). *SELL*, an adhesion molecule, regulates the transport of immune cells to lymphocyte aggregates in TME (53). Tumor cells may utilize *SELL* to facilitate their detachment from the primary tumor, thereby promoting tumor metastasis and dissemination. Research by Liao et al. has inferred that *SELL* is involved in regulating the generation of PNAd and MAdCAM-1+ HEVs in TLS (54). These studies indicate that *SELL* may have a pivotal function in the initial stage of TLS formation. Research on this aspect is still lacking currently.

*PRRX1*, named as paired related homeobox 1 and *PRX1*, works as a primary transcription factor of cancer-associated fibroblasts (CAFs) (55). Prior research has established a robust link between elevated *PRRX1* expression and the progression and recurrence of CRC, breast cancer, and esophageal cancer, leading to poor prognosis and drug resistance (55–57). Zhong et al. discovered that *PRRX1* promotes tumor cells to migrate and invade by targeting the IL-6/JAK3/STAT3 axis (58). Silencing of *PRRX1* may indirectly influence the proliferation and differentiation of TLS in CRC by inhibiting this axis (59). We checked the expression of *PRRX1* in the TLS+/- groups through IHC in this study. In summary, *PRRX1* may function as a therapeutic target for CRC treatment and be a negative immunomodulatory regulator of TLS.

Our research provided an innovative perspective for exploring the crosstalk between TLS and CRC TME. Nevertheless, our research still has some weaknesses. The data bias caused by retrospective studies is inevitable. TCGA and GEO cannot provide MRI imaging and hematological data. Furthermore, applying spatial transcriptomics to analyze TLS *in situ* may bring new insights.

## 5 Conclusion

Based on TRGs and DEGs, we discriminated the TLS related subclusters, constructed a prognostic risk model, and explored the potential mechanisms of hub genes to regulate TLS and CRC progression. Low-risk group exhibited a more favorable clinical outcome, richer immune infiltration, and better immunotherapy efficacy. Moreover, a preliminary exploration of the mechanisms of hub genes could help identify potential therapeutic targets.

## Data availability statement

The original contributions presented in the study are included in the article/**Supplementary Material**. Further inquiries can be directed to the corresponding author.

## Ethics statement

The studies involving human participants were reviewed by and approved by Institutional Ethics Committee of QuXian People’s Hospital. The participants provided their written informed consent to participate in this study. The studies were conducted in accordance with the local legislation and institutional requirements. The participants provided their written informed consent to participate in this study.

## Author contributions

ZX: Conceptualization, Data curation, Funding acquisition, Writing – original draft, Writing – review & editing. QW: Data curation, Investigation, Resources, Writing – original draft. YYZ: Conceptualization, Funding acquisition, Software, Supervision, Validation, Writing – original draft. XL: Conceptualization, Methodology, Project administration, Resources, Validation, Writing – original draft. MW: Conceptualization, Methodology, Project administration, Resources, Software, Writing – review & editing. YHZ: Conceptualization, Investigation, Methodology, Resources, Visualization, Writing – original draft. YP: Investigation, Methodology, Project administration, Writing – review & editing. KL: Data curation, Methodology, Software, Supervision, Writing – review & editing. MY: Investigation, Supervision, Validation, Writing – original draft. LL: Conceptualization, Supervision, Validation, Writing – review & editing. CW: Project administration, Software, Validation, Writing – review & editing. WW: Conceptualization, Funding acquisition, Investigation, Resources, Software, Supervision, Validation, Visualization, Writing – original draft, Writing – review & editing.
