# Supplementary material for: Exploiting tertiary lymphoid structures gene signature to evaluate tumor microenvironment infiltration and immunotherapy response in colorectal cancer
Source: Front Oncol. 2024 May 23;14:1383096. doi: 10.3389/fonc.2024.1383096 (PMC11153738; doi:10.3389/fonc.2024.1383096)

Supplementary Material

# Supplementary Figures and Tables

## Supplementary Tables

**Supplementary Table 1.** Clinical information of patients in TCGA-CRC, GSE38832, GSE17537 datasets.

**Supplementary Table 2.** The lists of DRGs, TCGA-TRGs, intersection DEGs, OS-DEGs, model genes.

**Supplementary Table 3.** Clinical information and IHC score of patients in QuXian cohort.

Supplementary Table 1-3 have been uploaded separately.

## Supplementary Figures

**Supplementary Figure 1.** Identification of TLS related subclusters with consensus clustering. **(A)** Cumulative distribution function (CDF) curves. **(B)** clusters connectivity matrix when k=2. **(C)** Delta area. **(D)** PCA demonstrated that all patients in TCGA-CRC cohort are well identified into two clusters when k=2. Patients proportion between two clusters of gender **(E)**, T stage **(F)**, N stage **(G)**, M stage **(H),** TNM stage **(I)**.


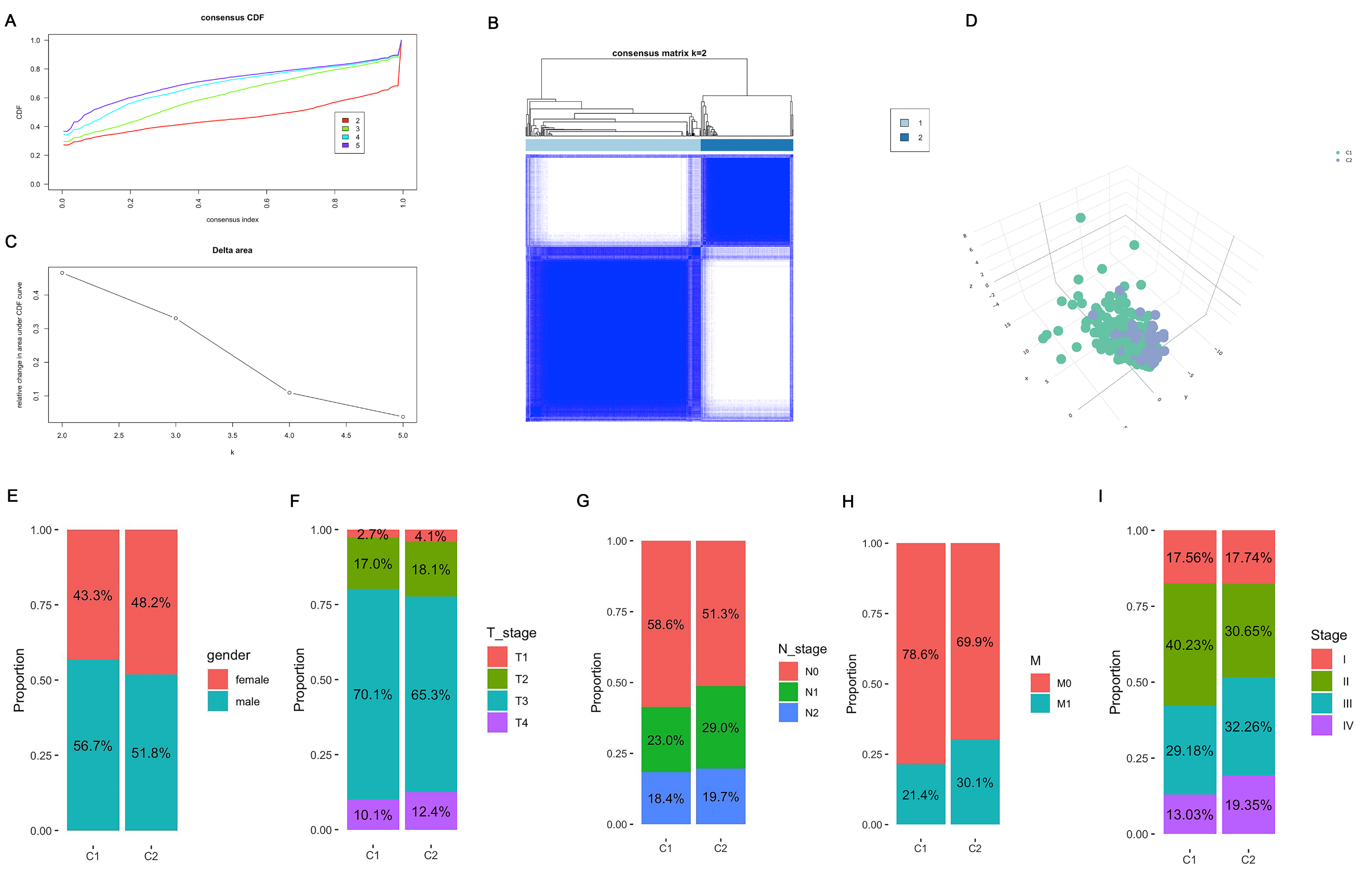


**Supplementary Figure 2.** Construction of the TLS-related risk model. **(A)** Volcanic map of DEGs between C1 and C2. **(B)** Volcanic map of DEGs between tumor and normal samples. **(C)** Venn diagram. **(D)** Lasso coefficient profiles. **(E)** Ten-time cross validation for the model parameter selection. The vertical line represented that 14 genes were finally identified. **(F)** Heatmap of model genes expression profiles.

**
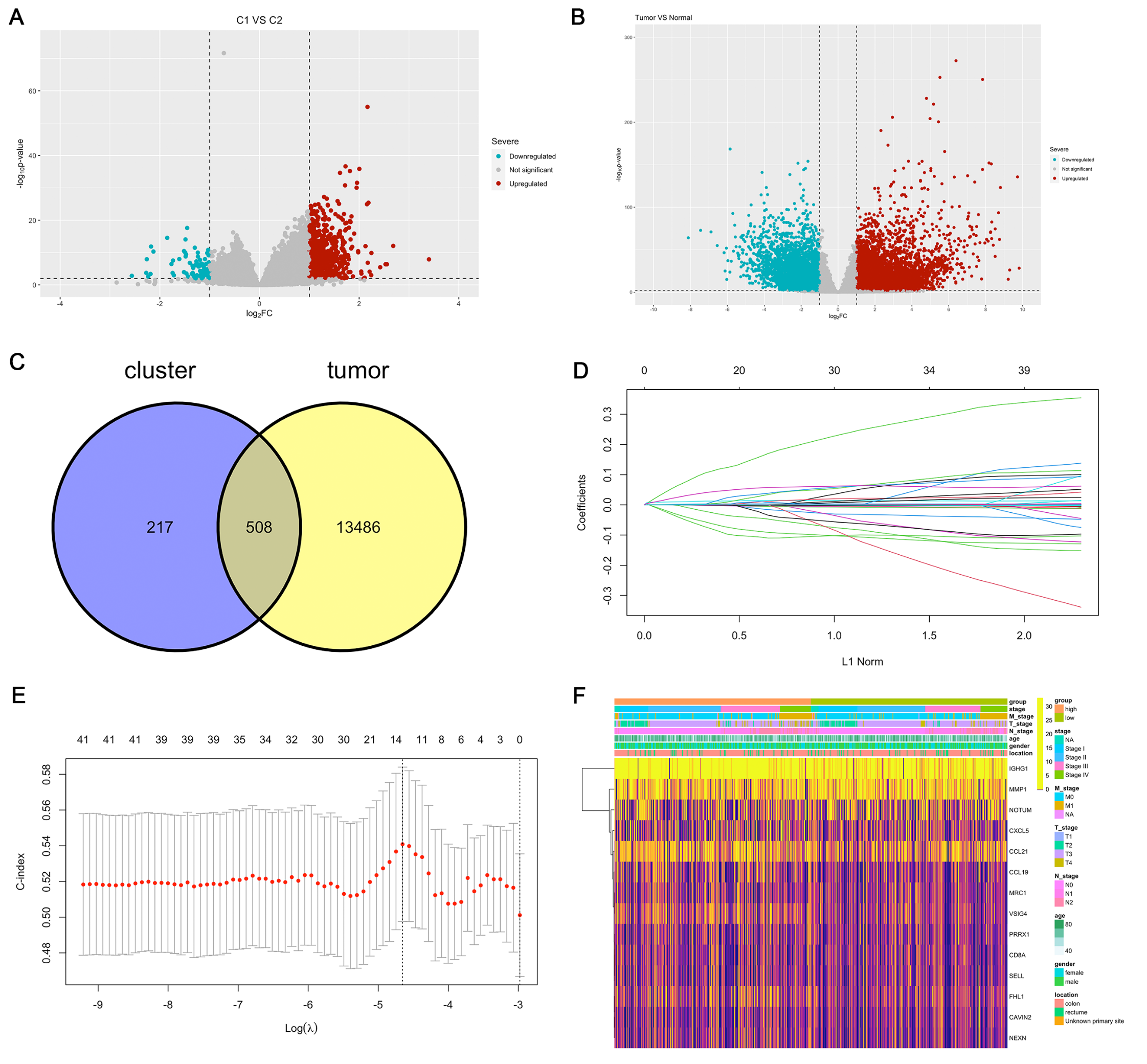
**

**Supplementary Figure 3.** The role of model in predicting immunotherapy response. Stroma **(A)**, immune **(B)** and TIME **(C)** scores between two risk groups. **(D)** TIDE score. **(E)** Survival analysis between risk groups in IMvigor210. **(F)** Distribution of the responders (people with CR/PR) and non-responders (people with SD/PD) between high and low-risk groups. Complete response (CR), partial response (PR), stable disease (SD), progressive disease (PD). *** p< 0.001.


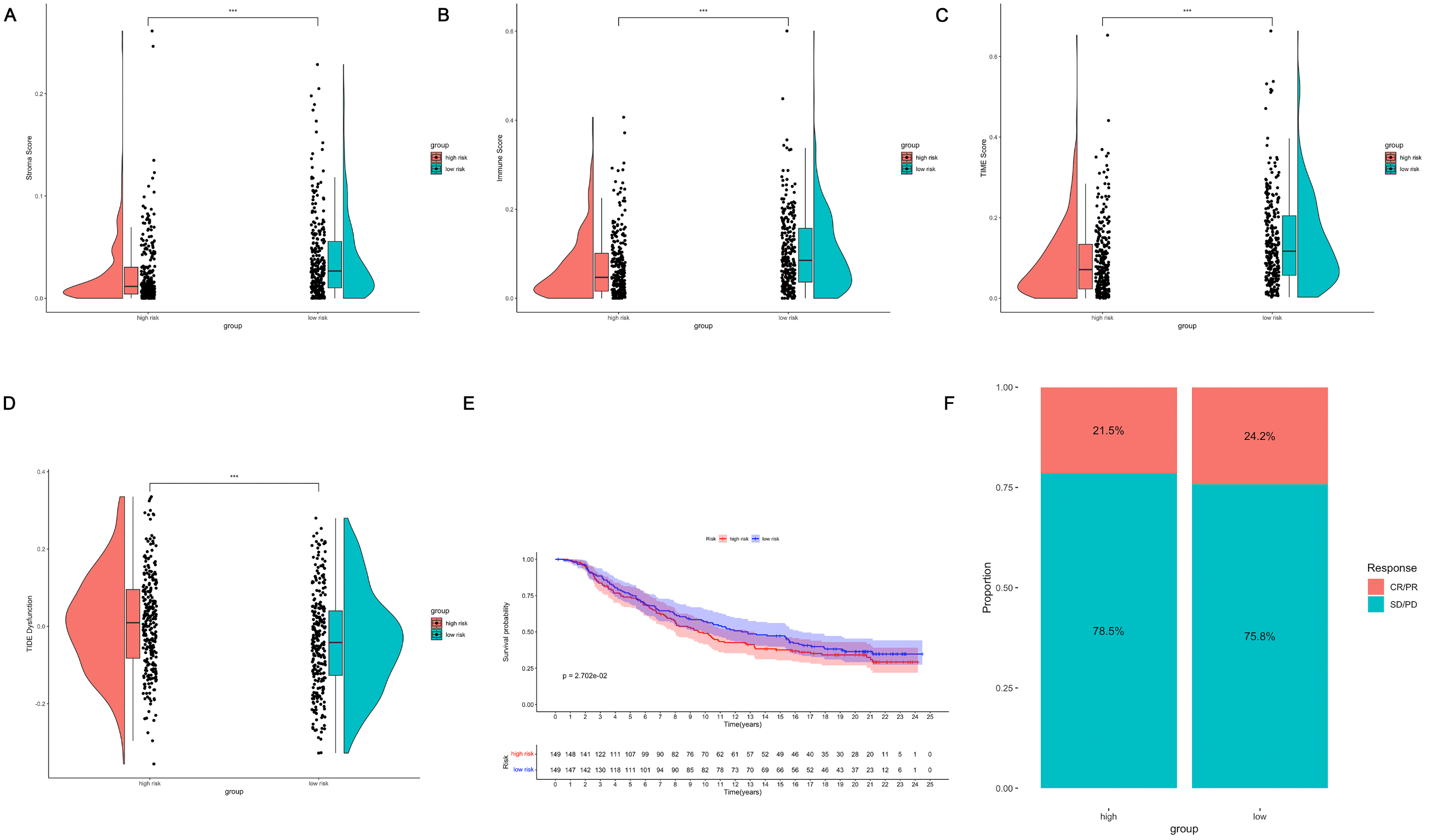


**Supplementary Figure 4.** Selecting of hub genes. **(A)** Genes relative importance ranking. **(B)** mRNA expression of VSIG4, SELL and PRRX1 in two risk groups **(C)** Correlation between hub genes and the other signature genes. **(D)** Bubble charm of the relationship between hub gene expression and immune infiltration.


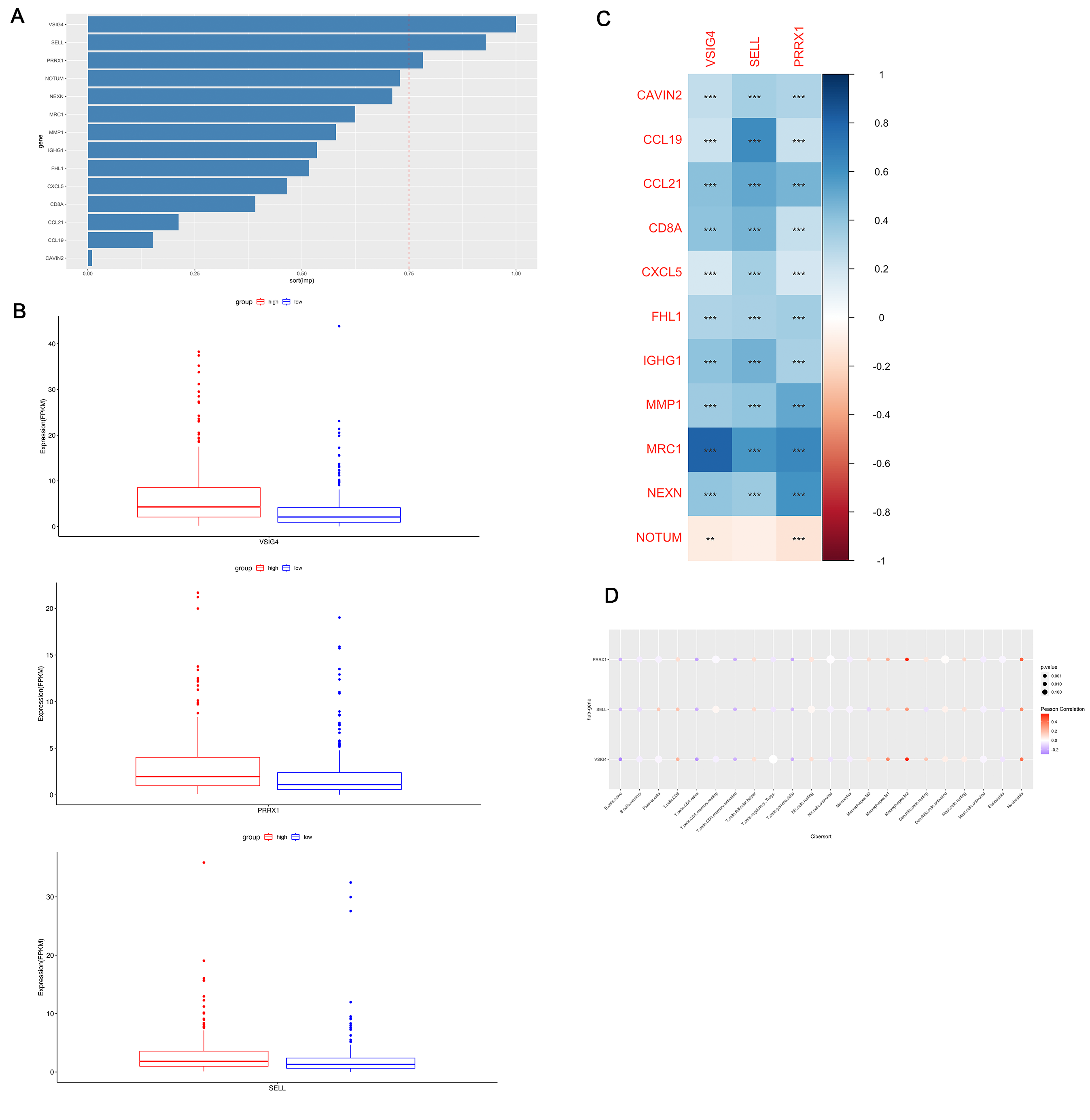

Supplement: Supplementary file 1 [file DataSheet_1.zip › Supplementary Material/Supplementary Material .docx]
