# Supplementary figures and images for: Exploiting tertiary lymphoid structures gene signature to evaluate tumor microenvironment infiltration and immunotherapy response in colorectal cancer
Source: Front Oncol. 2024 May 23;14:1383096. doi: 10.3389/fonc.2024.1383096 (PMC11153738; doi:10.3389/fonc.2024.1383096)

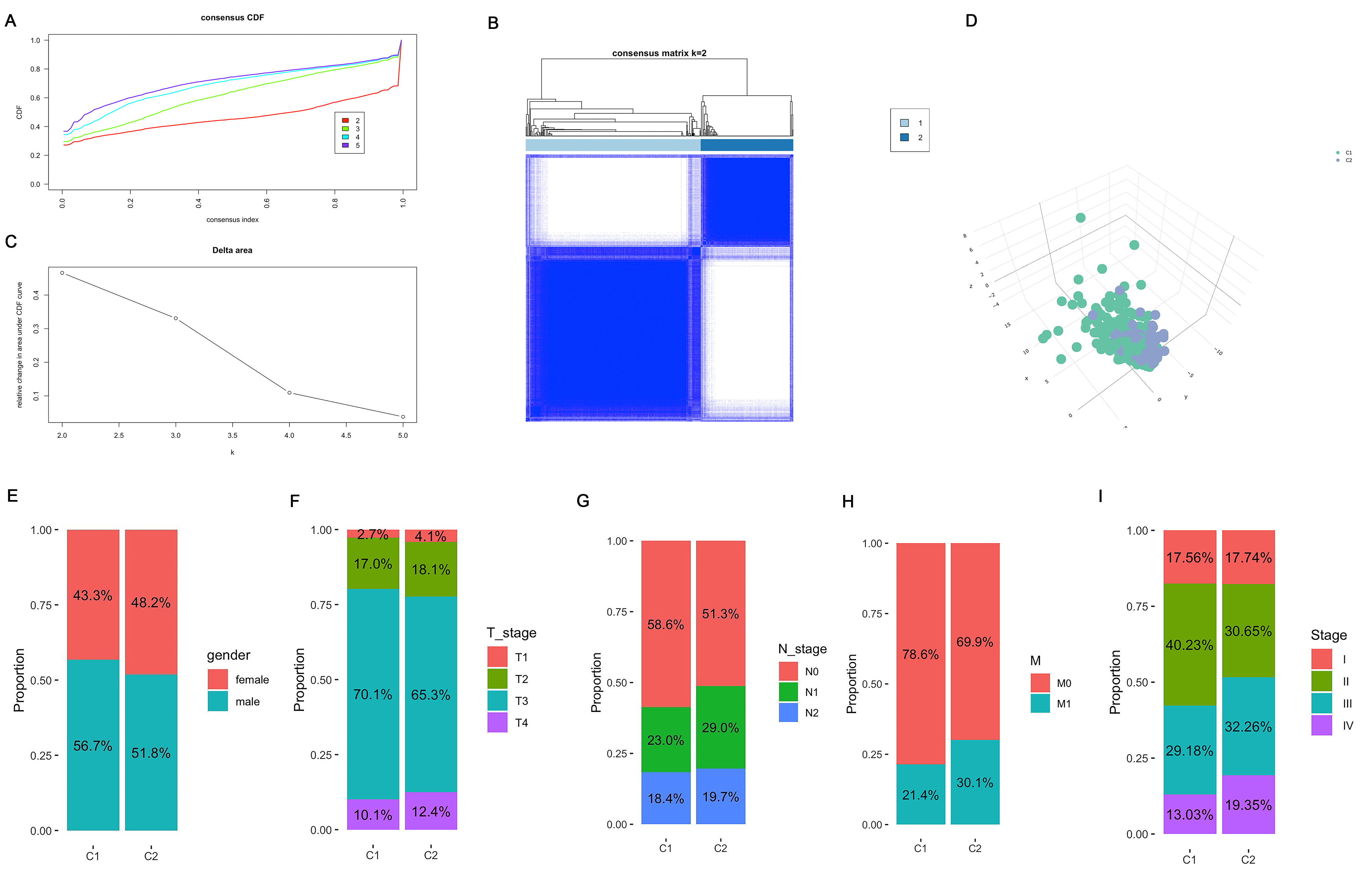

Supplement: Supplementary file 1 [file DataSheet_1.zip › Supplementary Material/Supplementary figure 1.tif]

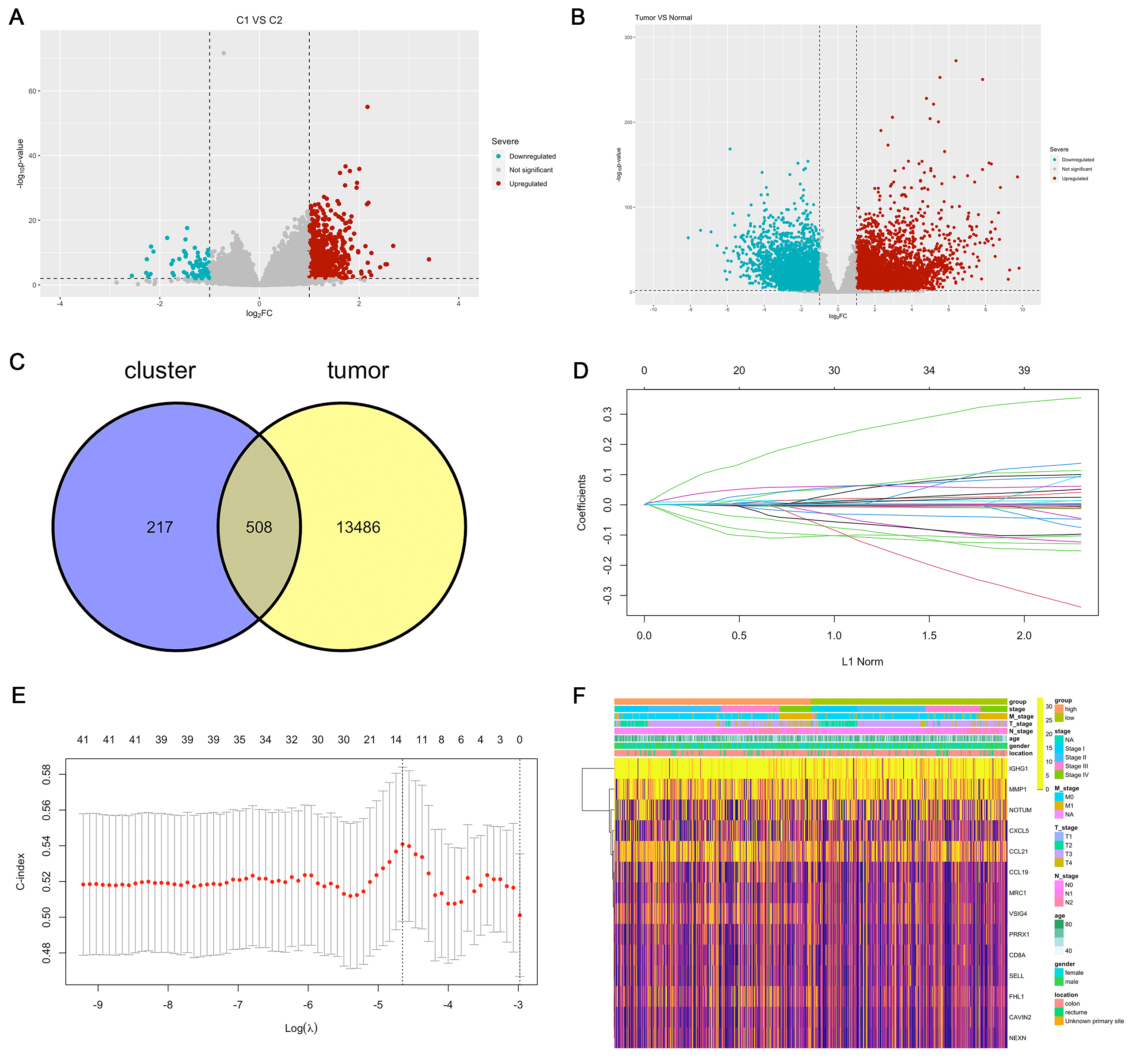

Supplement: Supplementary file 1 [file DataSheet_1.zip › Supplementary Material/Supplementary figure 2.tif]

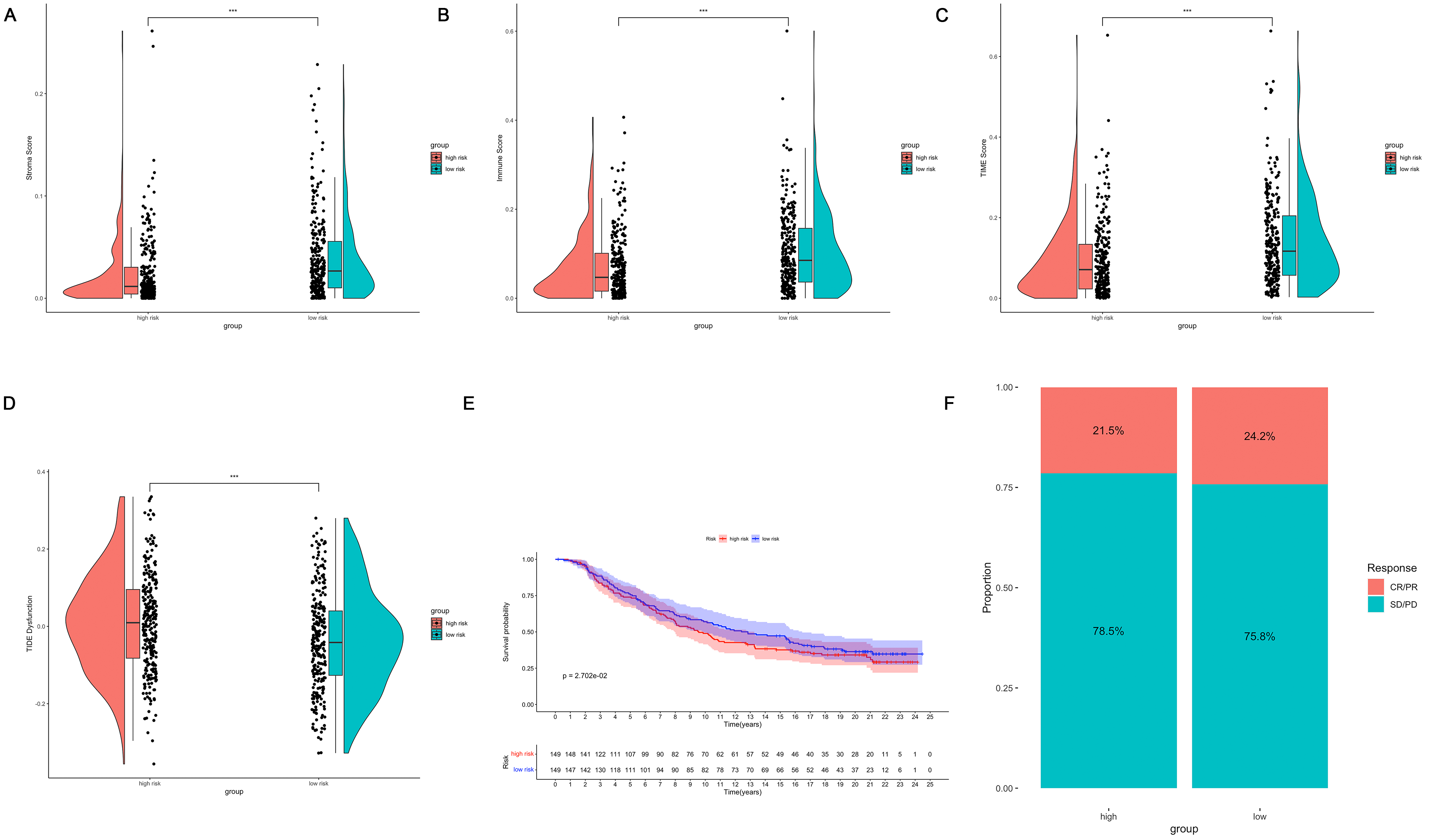

Supplement: Supplementary file 1 [file DataSheet_1.zip › Supplementary Material/Supplementary figure 3.tif]

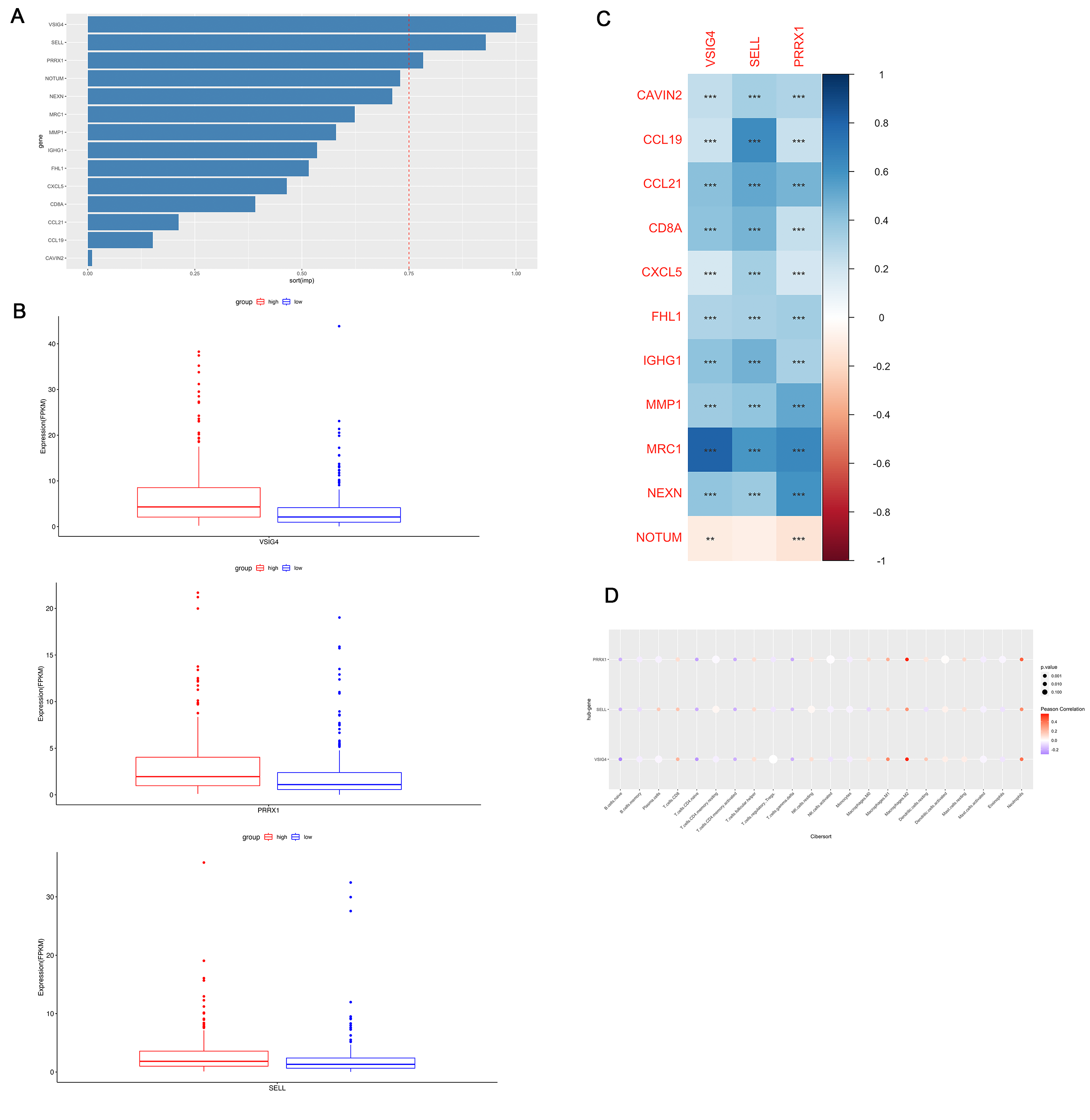

Supplement: Supplementary file 1 [file DataSheet_1.zip › Supplementary Material/Supplementary figure 4.tif]
